# Optimization of filtering criterion for SEQUEST database searching to improve proteome coverage in shotgun proteomics

**DOI:** 10.1186/1471-2105-8-323

**Published:** 2007-08-31

**Authors:** Xinning Jiang, Xiaogang Jiang, Guanghui Han, Mingliang Ye, Hanfa Zou

**Affiliations:** 1 National Chromatographic R&A Center, Dalian Institute of Chemical Physics, The Chinese Academy of Sciences, Dalian 116023, China

## Abstract

**Background:**

In proteomic analysis, MS/MS spectra acquired by mass spectrometer are assigned to peptides by database searching algorithms such as SEQUEST. The assignations of peptides to MS/MS spectra by SEQUEST searching algorithm are defined by several scores including Xcorr, ΔCn, Sp, Rsp, matched ion count and so on. Filtering criterion using several above scores is used to isolate correct identifications from random assignments. However, the filtering criterion was not favorably optimized up to now.

**Results:**

In this study, we implemented a machine learning approach known as predictive genetic algorithm (GA) for the optimization of filtering criteria to maximize the number of identified peptides at fixed false-discovery rate (FDR) for SEQUEST database searching. As the FDR was directly determined by decoy database search scheme, the GA based optimization approach did not require any pre-knowledge on the characteristics of the data set, which represented significant advantages over statistical approaches such as PeptideProphet. Compared with PeptideProphet, the GA based approach can achieve similar performance in distinguishing true from false assignment with only 1/10 of the processing time. Moreover, the GA based approach can be easily extended to process other database search results as it did not rely on any assumption on the data.

**Conclusion:**

Our results indicated that filtering criteria should be optimized individually for different samples. The new developed software using GA provides a convenient and fast way to create tailored optimal criteria for different proteome samples to improve proteome coverage.

## Background

Because of the high sensitivity, mass spectrometry has been widely used for protein identification and characterization in proteome researches within the past decade[1,2]. Shotgun proteome approach, which is based on analysis using liquid chromatography coupled with tandem mass spectrometry (LC-MS/MS), can be applied to analyze complex protein mixtures directly even without any prior purification step. Large-scale proteome profiling using multidimensional LC-MS/MS has become increasingly applied for the analysis of many biological samples, including various mammalian tissues, cell lines, and serum/plasma [3-8]. In shotgun proteomics, complex protein mixtures are first digested by the enzyme (e.g. trypsin) to produce peptide mixtures. Then the peptide mixtures are subjected to extensive separations such as strong cation exchange chromatography (SCX) coupling with on-line or off-line reversed-phase capillary LC (RPLC). Peptides eluting from the reversed phase capillary LC column are sprayed into tandem mass spectrometer to produce MS/MS spectra. And then peptide sequences are assigned to experimental MS/MS spectra by database searching algorithm.

SEQUEST[9], Mascot[10] and other database searching algorithms match experimental spectra with theoretical spectra which are generated from peptide sequences in silico, and then calculate scores to evaluate how well they match. These scores help discriminating between correct and incorrect peptide assignments. One of the major issues in database search for proteome analysis is to determine the false-discovery rate (FDR) of the identifications. FDR is the rate at which significant identifications are actually null[11]. A variety of methods were developed to determine FDR for peptide identifications. Some efforts have been made on establishing statistical analysis methods [11-17] to determine the possibility of positive identifications, e.g. PeptideProphet[12]. Complicated statistical algorithms are often needed in these methods. Another simpler way to evaluate FDR is using decoy proteome approach which was introduced by Peng et al[18]. Determination of FDR in this method is based on the database searching using a composite database including original protein database and its reversed version. Statistically, the probability that a peptide is identified incorrectly from reversed database is expected to be same as the probability that it is identified incorrectly from original protein database as the sizes of reversed database and original database are the same [19-21]. Therefore, FDR can be calculated using the following equation:

FDR = 2*n(rev)/(n(rev)+n(forw)),

where n(forw) and n(rev) are the number of peptides identified in proteins with forward (original) and reversed sequences, respectively[18,22]. The database searching strategy using composite database is also known as reversed database searching strategy. Because of the simple usage, it has been widely used in the evaluation of proteomic search results[18,22-26] including post-translation modification (PTM) researches[19,27,28].

SEQUEST[9] is one of the commonly used database searching algorithms. It first counts the peaks which are common in experimental and theoretical spectra, and computes a preliminary score (Sp). Then it selects a proportion of top candidate peptides based on the rank of preliminary score (Rsp) for cross-correlation analysis. So, for each candidate peptide identification, several scores and rankings are determined. To distinguish correct identifications from incorrect identifications, filters using a set of database searching scores are applied, including two commonly used scores, Xcorr and ΔCn. In order to evaluate FDR of the identifications, reversed database searching could be performed and the FDR could be determined by Equation (1). To control FDR, many research groups usually use fixed Xcorr values and manually increase ΔCn to get peptide identifications with specific FDR[25], or use a fixed ΔCn value and manually increase of Xcorr scores [18]. However, these new criteria which were determined by adjusting only one score filter to reach a specific FDR may be not optimal.

Genetic algorithm (GA) belongs to evolutionary algorithms and applies natural selection process, where better fitted species are selected. The optimization process of this algorithm is based on multi-point-search for which many solutions are calculated simultaneously[29]. If the fitness function is properly designed, GA has the ability to search through very large sets of possible solutions and converge to an optimal or near optimal solution quite quickly. It has been successfully applied to process MS data in proteome researches [30-32].

In this work, we combined the decoy database searching approach with automated filter criteria optimization, and developed a software suite named SFOER (SEQUEST Filter Optimizer Using Genetic Algorithm) using GA which enables simultaneous optimization of multiple SEQUEST score filtering criteria. The optimized criteria were used to filter datasets which were generated from two different human samples and resulted in approximate 20% increase of peptide identifications than that using conventional criteria[14,25,33] while FDR were kept the same (<1%). Direct comparison between SFOER and PeptideProphet has been performed using both complex human samples and standard protein mixtures. Compared with PeptideProphet, SFOER showed nearly same ability in distinguishing correct peptide identifications from incorrect ones with only 1/10 of the processing time. And because SFOER doesn't rely on models which are based on possible unfounded assumptions, it provides a safe way for fast determination of tailored optimal filtering criteria for different proteome samples, thus, higher proteome coverage can be achieved.

## Results and discussion

To evaluate the confidence of peptide assignments by SEQUEST and generate the score distribution for peptide identifications, we have generated large datasets of human proteome samples by SCX/RPLC-MS/MS[34]. Approximately 277,000 MS/MS spectra were generated from human liver tissue lysate. All MS/MS spectra were searched by SEQUEST against a composite database containing human IPI proteins in both forward and reversed orientation. Herein only the top matched peptide from a spectrum with specific charge state was accepted. Approximate 11,000, 186,000 and 181,000 peptides according to the charge states of 1+, 2+ and 3+ were finally generated. 165,966 (43.86%) peptides were derived from reversed protein database and 212,430 (56.14%) were from forward protein database.

### True and false assignment distribution

**Figure 1:**
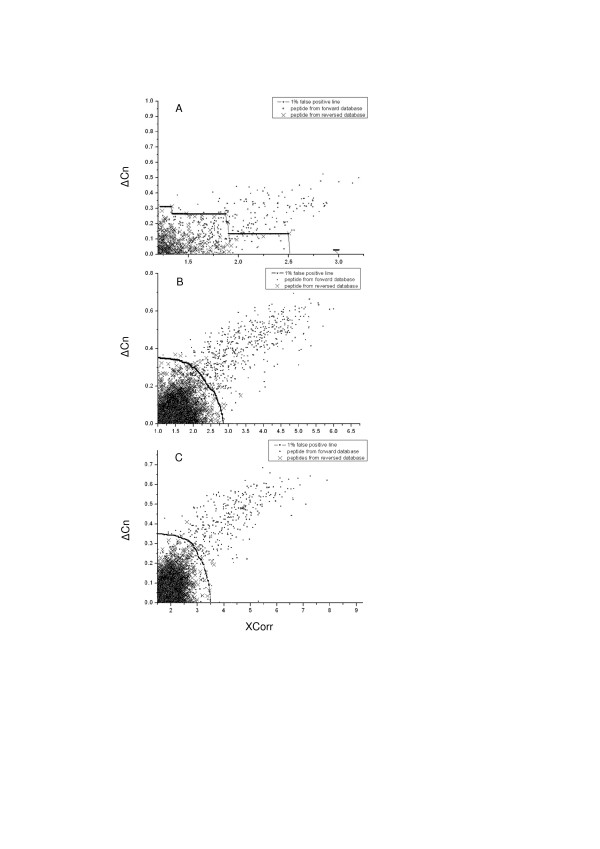
**Distribution of peptides identified from human liver tissue lysate by SEQUEST**. A) Singly charged peptides; B) Doubly charged peptides; C) Triply charged peptides. Each data point represents a peptide identification from the composite database: cross represents peptide identification from reversed sequence while square indicates peptide identification from forward sequence. Cumulate curves drawn in each graph are 1% false-discovery curves. Each point on curves indicates a filtering criterion leading to peptide identification with FDR of 1%, and the identified peptides by each criterion present in the region where Xcorr and ΔCn scores are higher than the Xcorr and ΔCn cutoffs in each set of criteria. Graphs were drawn using the Speed Model by Origin 7.5 with 5000 max points per curve, and three raw graphs with all data points were shown [see Additional file 1].

To investigate whether it was necessary to optimize the filtering criteria after SEQUEST database search, we first investigated the distribution of peptides identified from forward protein database and reversed protein database. The distributions of peptides with different Xcorr and ΔCn values are shown in Figure 1A, 1B and 1C according to the charge states of 1+, 2+ and 3+, respectively. Evidently, peptides with reversed sequences (represented as crosses) centralized at the region with low Xcorr and ΔCn scores, which indicated that peptide assignments with SEQUEST scores lying in this region were more likely to be random matches. And in the region where Xcorr scores were high enough, there was nearly no peptide with reversed sequence, and ΔCn scores of peptides in this region were always high. Therefore, peptides which were identified with high Xcorr and ΔCn scores were more likely to be true assignments.

To obtain confident identifications with specific FDR (< 1%), filter criteria with two SEQUEST scores, Xcorr and ΔCn, need to be adjusted. A series of filtering criteria using these two cutoff scores can be determined in this way: Xcorr cutoff scores were increased by a specific value (e.g. 0.05) step by step, and ΔCn cutoff scores were decided accordingly with the Xcorr cutoff values for the aim that identifications passed the filtering criterion had an overall FDR less than 1%. Cumulate curves of these filters determined above were shown in each graph of Figure 1 according to the charge states of 1+, 2+ and 3+, and every point on each curve indicated a set of criteria leading to FDR < 1% for peptide identifications with a specific charge state. These curves indicated that to achieve peptide identifications with FDR less than 1%, various criteria can be used.

**Figure 2:**
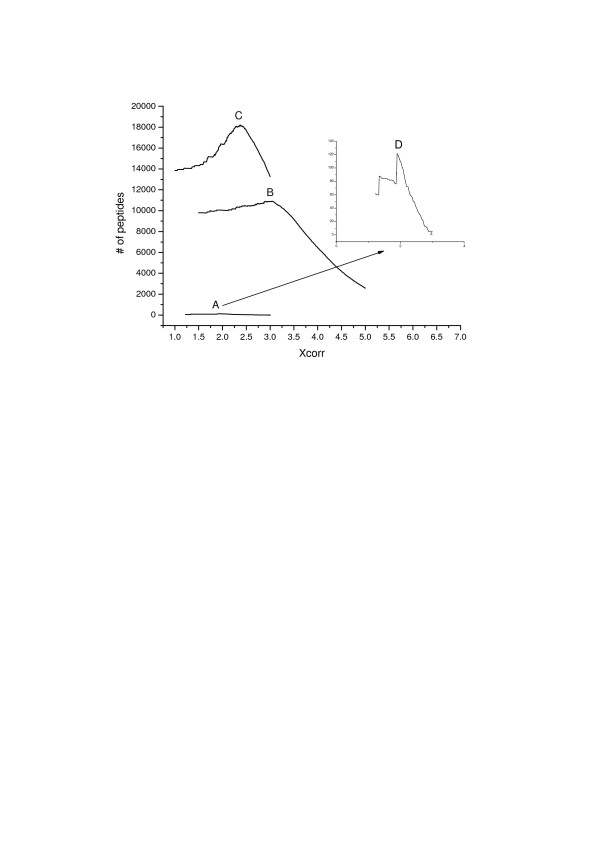
**Relationship between the number of peptide identifications and Xcorr values in different criteria which leaded to these identifications for human liver tissue lysate at same FDR (<1%)**. To achieve less than 1% FDR, ΔCn cutoff for each criterion changes with the Xcorr cutoff. Curves for three different charge states were drawn separately: A) for singly charged peptides, B) for triply charged peptides and C) for doubly charged peptides. D) is the zoomed curve for singly charged peptides.

To demonstrate the dependence of the number of identified peptides on the application of different filter criteria at same FDR (<1%), relation between the number of identified peptides and Xcorr cutoff values in different criteria which result in these identifications is shown in Figure 2. Evidently, the number of peptide identifications changed greatly when criteria with different Xcorr cutoffs were used. For example, as shown by curve C in Figure 2, the number of identified doubly charged peptides was 18,218 when criterion with Xcorr cutoff value of 2.37 was used, but this number changed to 15,261 when criterion with Xcorr value of 2.8 was used. Approximately 20% difference in number of peptide identifications between these two sets of criteria was observed. In addition, to reach FDR < 1%, ΔCn cutoff values of these two criteria were 0.213 and 0.069, respectively. According to above results, simultaneous optimization of different SEQUEST score combinations for filtering criteria can result in more positive peptide identifications and reduce false-negative detections which are true assignments but may be rejected by conventional filtering criteria while confidence levels keep the same.

### Optimization of filtering criteria by genetic algorithm

**Figure 3:**
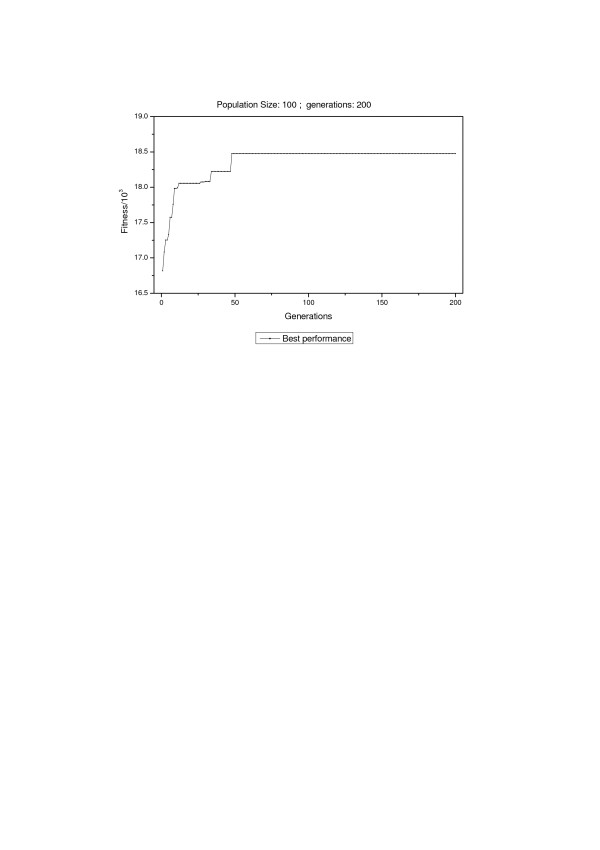
**Dependence of fitness on generations for doubly charged peptides**. Fitness for each individual (criterion) represents the number of peptide identifications filtered by this criterion. Fitness of the fittest individual in each generation was represented as black dots.

Our aim of this study was to develop an approach to optimize the filtering criteria which maximized the number of peptide identifications without increase of FDR. To achieve this purpose, genetic algorithm[29] was used to develop a Java software suite named SFOER which took SEQUEST scores such as Xcorr, ΔCn etc. as its weights and number of peptide identifications as its scale. Fitness was calculated using equation (2). The optimization was composed of 200 generations. Each generation had 100 individuals, and these individuals were of three types, one came from parents, another which contained combined information from two parents was generated by the cross-over of individuals in current generation, and the third was the "mutant" one which contained new introduced information. Probabilities of mutation and cross-over were set to 0.01 and 0.2, respectively. Each individual contained four genes including Xcorr, ΔCn, Sp and Rsp. Limit of FDR was set to 1%. After optimization, the resulting fittest individual was considered to be the optimized set of weights. Since the distributions of SEQUEST scores for different charge states are different as shown in Figure 2, optimization of filtering criteria for different charges states were conducted independently. Details of criterion optimization for peptides with double charge state are represented in Figure 3. As the convergence was obtained over the first 100 generations, we allowed GA to continue to evolve another 100 generations for further improvement of the optimization.

On the basis of above GA optimization procedure, SFOER was utilized to optimize the filtering criteria for dataset generated from human liver tissue lysate. Finally, we got the following optimized criteria (FDR <1%): according to the charge states of 1+, 2+ and 3+, Xcorr scores should be bigger than 1.76, 2.31 and 2.41, ΔCn should be bigger than 0.061, 0.199 and 0.265, Sp should be bigger than 44.42, 104 and 276.9 and Rsp should be within 3, 4 and 2. Filtered by this set of criteria, 29,934 positive peptides were generated, including 162 singly charged peptides, 18,513 doubly charged peptides and 11,259 triply charged peptides.

To evaluate the performance of this set of optimized filtering criteria, we then compared it with the conventional criteria[14,25,33]. Similar to them, criteria were determined as follows: Xcorr cutoffs were set as 2.0, 2.5 and 3.8 for singly, doubly and triply charged peptides and ΔCn cutoff was determined by the increase of its value until FDR for peptide identifications was less than 1%. Finally the ΔCn cutoff was determined to be 0.164 for the human liver tissue dataset. When above criteria were applied, 99, 17,827 and 7,385 singly, doubly and triply charged peptides were identified. Using the filtering criteria optimized in this study, there was an 18.27% increase of peptide identifications (29,934 vs 25,311) with same FDR (<1%) after combining the results of all charge states. And 15.11% more unique peptides (5285 vs 4591) were generated by the optimized criteria (Table 1).

**Table 1:** Comparison of the performance of conventional criteria, PeptideProphet and SFOER in peptide identifications for the analysis of human liver tissue lysate^a^

	Conventional criteria^b^	PeptideProphet^c^	SFOER^d^
# 1+	99	26	162
# 2+	17950	17451	18606
# 3+	7388	12587	11313
# total	25428	30064	30081
%incr	/	18.2%	18.3%
# false pep	126	113	147
FDR	0.99%	0.75%	0.98%
#unique pep	4591	5175	5285
%incr unique pep	/	12.7%	15.12%
# proteins	1467	1596	1665

a. Summary of each category returned by different strategies: #1+, #2+ and #3+ indicates the number of peptide identifications for charge states of 1+, 2+ and 3+ respectively. #total = (#1+) + (#2+) + (#3+). #false pep indicates the number of peptides from reversed database, while #unique pep is the number of total unique peptides. Increase of peptide identifications (%incr) and unique peptide identifications (%incr unique pep) by SFOER and PeptideProphet are shown. #proteins are the number of positive proteins identified by the strategies. FDR of identifications are also shown.

b. Conventional criteria. Xcorr > 2.0, 2.5 and 3.8 for singly, doubly and triply charged peptides, respectively and ΔCn > 0.164 for all charge states [25, 33].

c. Cutoff is set as PeptideProphet probability > 0.9 [13, 36-38].

d. Optimized criteria determined by SFOER are: according to the charge states of 1+, 2+ and 3+, Xcorr scores > 1.76, 2.31 and 2.41, ΔCn > 0.061, 0.199 and 0.265, Sp > 44.42, 104 and 276.9 and Rsp < 3, 4 and 2.

When the optimization was performed on another very different sample, human blood plasma, different set of optimal filtering criteria was generated. According to the charge states of 1+, 2+ and 3+, Xcorr scores should be bigger than 1.88, 2.31 and 2.40, ΔCn should be bigger than 0.179, 0.27 and 0.319, Sp should be bigger than 238, 71 and 215.6 and Rsp should be within 80, 2 and 1. Filtered by this set of criteria, 14,218 peptides were generated. And there was an 15.3% increase of peptide identifications than those resulted from conventional criteria (Xcorr cutoffs bigger than 2.0, 2.5 and 3.8 for singly, doubly and triply charged peptides and ΔCn scores bigger than 0.265)[14,25,33].

Evidently, optimized criteria for datasets from these two different human samples were different even with same separation and analysis conditions (Table 2). While the optimized Xcorr cutoffs for datasets from these two samples were almost the same (1.76, 2.31, 2.41 vs 1.88, 2.31, 2.40), other score and ranking cutoffs were quite different: according to the charge states of 1+, 2+ and 3+, these cutoffs for human liver tissue were ΔCn > 0.061, 0.199 and 0.265, Sp > 44.42, 104 and 276.9 and Rsp rankings should be within 3, 4 and 2; but for human blood plasma, these cutoffs were ΔCn > 0.179, 0.27 and 0.319, Sp should be bigger than 238, 71 and 215.6 and Rsp should be within 80, 2 and 1. Similar phenomena have also been observed by Smith and Colleagues[35], and they attributed this to the different protein abundances in different samples.

**Table 2:** The optimized criteria of peptide identifications from human liver tissue lysate and human plasma by SFOER with FDR less than 1%

	Charge	Xcorr	ΔCn	Sp	Rsp
liver tissue	1+	1.76	0.061	44.42	3
	2+	2.31	0.199	104	4
	3+	2.41	0.265	276.9	2
plasma	1+	1.88	0.179	238	80
	2+	2.31	0.270	71	2
	3+	2.40	0.319	215.6	1

In most cases, the differences on proteome analysis were inevitable: protein samples may come from different tissues or even different species, mass spectra may be collected by different type of mass spectrometers under different separation conditions and so on. These differences will result in the generation of datasets with different characteristics. Statistical approaches based on training with some assumptions on one type of dataset may only work well on datasets with that particular type. However, for other type of datasets with different characteristics, these approaches may need retraining or redesign. While SFOER does not employ any statistical method and no training was required. So SFOER can be applied to process any database search results as long as the searches were performed against decoy database where FDR could be easily determined. By using this GA based software suite, optimized criteria for different datasets can be easily determined, and these tailored optimal criteria should be very effective to improve the coverage for proteome analysis.

Discrimination powers of the filtering criteria optimized by SFOER with different combinations of SEQUEST scores were also evaluated. The numbers of peptide identifications from liver tissue sample by applying these filtering criteria are shown in Table 3. For filtering criteria using three cutoff scores, the one using Rsp, Xcorr and ΔCn yielded more peptide identifications than that using Sp, Xcorr and ΔCn. And the number of identified peptides by using the first set of criteria was very close to that obtained by the optimized criteria using all four scores. Thus, Sp scores had less important contribution to the discrimination than Rsp did. Compared to the optimized criteria using all four scores, criteria using only two commonly used scores were also effective in reducing false-negative peptide identifications as only 3.35% decrease of peptide identifications were observed. This indicated that commonly used criteria which consisted of two cutoff scores, Xcorr and ΔCn, were effective in proteome researches, but if wanting to get higher proteome coverage, optimized criteria using other SEQUEST scores and rankings were needed.

**Table 3:** Summary of the peptide identifications from human liver tissue by applying filtering criteria optimized using different score combinations

	All four scores	Xcorr ΔCn Rsp	Xcorr ΔCn Sp	Xcorr ΔCn
# peptides	30,081	29,996	29,595	29,248
# increase	2.87%	2.56%	1.19%	/
FDR	0.977%	0.980%	0.980%	0.998%

### Classification performance of SFOER

**Figure 4:**
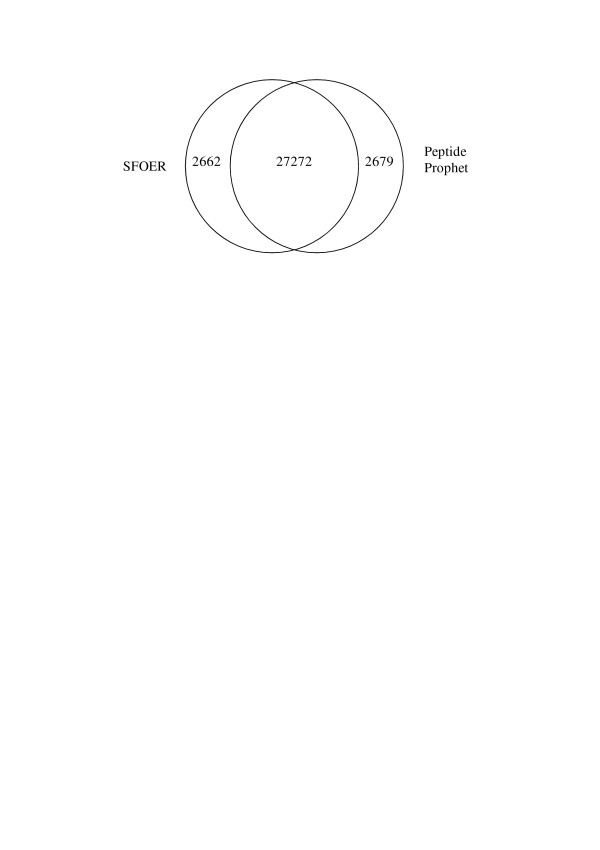
**Overlap of peptides identified by SFOER and PeptideProphet for human liver tissue lysate**. The numbers of peptide identifications by one or both algorithms are indicated, e.g., 27,272 peptides are identified by both algorithms (intersection).

PeptideProphet is a statistical approach, based on the expectation maximization algorithm (EM), for validation of peptide identifications made by tandem mass spectrometry and database searching[12]. Database search results for human liver and plasma samples were also processed by PeptideProphet. Probability thresholds of 0.9 were set for which empirical error rates for these two datasets were 1.1% and 1.2% respectively[13,36-38]. And the corresponding FDR were determined as 0.75% and 1.13% for these two datasets by employing reversed database searching strategy. There were 29,951 and 14,101 peptides identified by PeptideProphet for liver tissue sample and plasma sample, respectively. Compared with PeptideProphet, the numbers of peptides identified for the two human proteome samples by SFOER were nearly the same (29,934 vs 29,951 for liver tissue sample and 14,218 vs 14,101 for plasma sample). There was 91.2% overlap of the peptide identifications between PeptideProphet and SFOER, which means majority of the identified peptides were same for both approaches (Figure 4). Detail comparison of the performances on human liver lysate between conventional criteria, PeptideProphet and SFOER is shown in Table 1. The total numbers of identified proteins are also given in Table 1. Because of the increase of peptide identifications, the protein identifications also increased obviously when SFOER was used.

Compared with the conventional approach, the numbers of identified peptides increased significantly when the filtering criteria optimized by SFOER were applied. A concern for this is that whether the increased peptide identifications are true identifications. For datasets from human liver tissue sample, 5,588 extra peptide identifications were achieved when the filtering criteria optimized by SFOER were applied. It is impossible to manually validate all of these peptide identifications. A practical way is to randomly select small portion of the increased peptide identifications and manually check with their spectra. Thus 300 out of from 5,588 extra peptides identifications were randomly selected. Each of these spectra was assessed for acceptable signal-to-noise ratio and the presence of at least three consecutive b or y ion fragments[39]. Finally 98.3% (295 out of 300) of these peptides were true positive and the false-discovery rate was very close to the overall predicted FDR. It was found that 84% (4,693 out of 5,588) of the increased peptides can also be detected by PeptideProphet at a probability cutoff of 0.9 for which the empirical error rate was 1.1%. Above results clearly demonstrated that the additional peptide identifications obtained by SFOER were quite confident. (MS/MS spectra of the increased peptide identifications using our optimized criteria can be downloaded from our website[40]).

Classification performance of SFOER was further validated by standard protein mixture. Tryptic digest of seven standard proteins was selected as the sample. And the acquired MS/MS spectra were searched against a composite database containing both forward and reversed sequences of all control proteins (including trypsin) as well as forward and reversed protein sequences from yeast, chosen for its low homology with readily available control proteins. Because the proteins present in the sample were known, correct and incorrect peptide assignments can be easily distinguished by the rule whether it is from known standard proteins. Thus actual FDR, i.e. the observed FDR, can be determined by the percentage of peptide identifications not from standard proteins among all peptide identifications, while predicted FDR was determined by Equation (1). If not otherwise stated, FDR refers to the predicted FDR. The classification performance of SFOER could be evaluated by comparing the actual and predicted FDR.

**Figure 5:**
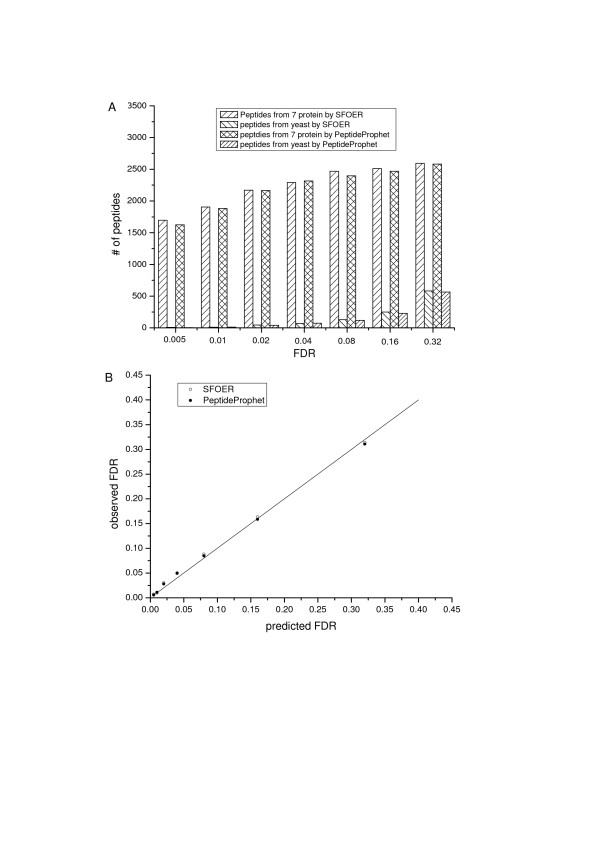
**Evaluation of the classification performances of SFOER and PeptideProphet with standard protein mixture**. A) Number of correct and incorrect peptide identifications by SFOER and PeptideProphet under different FDR, where incorrect peptide identification indicates peptide assignment from forward yeast database while correct one is from known standard proteins and trypsin. B) Predicated and observed FDRs. Observed FDR is calculated as the number of peptide identifications not from standard proteins over total peptide identifications, while predicated FDR is calculated using equation (1). Observed FDR for SFOER are presented by open circles, while observed FDR for PeptideProphet are represented by filled circles.

LC-MS/MS analyses of 7 standard protein mixture digest resulted in a collection of 105,000 spectra. Performance of SFOER was also compared with that of PeptideProphet using this standard protein dataset. A series sets of filtering criteria were optimized by SFOER with FDR increased from 0.005 to 0.32. Then peptide identifications with different confidence levels were generated by utilizing these optimized criteria. For PeptideProphet, manual adjustment of the probability threshold was used to generate peptide identifications with different FDR. The number of correct peptide identifications (peptide from standard proteins) and the number of incorrect peptide identifications (peptide from forward protein sequences in yeast database) are shown in Figure 5A. With the increase of FDR, SFOER showed nearly same performance with PeptideProphet except a slight improvement in the number of correct peptide identifications. And PeptideProphet showed a small increase of power in trading-off incorrect peptide identifications. Plot in Figure 5B are the observed FDR as function of the predicted FDR. It can be seen that the observed and predicted FDR matched very well for both SFOER and PeptideProphet. However, small increases of observed FDR were found for both cases. This probably because that our evaluation method didn't take commonly contaminants such as keratins into account. On the basis of above results, reversed database searching algorithm essentially provided a reasonable estimation of the actual error. The optimization by SFOER based on reversed database strategy was reasonable and FDR of peptide identifications evaluated by reversed database strategy can essentially reflect the actual FDR.

GA is a very efficient algorithm and is widely used in searching for optimal or near optimal solutions. Thus, SFOER which employing GA should inherit this advantage. Approximately 277,000 spectra (12 LC-MS/MS runs) were processed by PeptideProphet and SFOER on a Pentium 4 (3.0 GHz) computer separately. The optimization procedure using SFOER took less than 4 min (10 s for 1+, 100 s for 2+ and 99 s for 3+), while the procedure for calculation of probability by PeptideProphet took about 38 min. And the IO procedures (for PeptideProphet, it consisted of assembling peptides from out files to html files and the conversion of files from html format to xml format, while for SFOER it only included the assembling of peptides from out files to plain text files) took about 40 min and 28 min for PeptideProphet and SFOER, respectively. Evidently, SFOER was much faster than PeptideProphet for which only 1/10 of time was needed for the searching of optimal criteria (without consideration of IO procedures).

For model based algorithm like PeptideProphet, accuracy relies on the fitness between the empirical model and obtained datasets. If the model accurately reflects the physical processes by which the data are generated, it can work well even for a small amount of training data. On the other hand if the data distributes in a significant way, classification errors proportional to the degree of divergence result. However, SFOER is less risky for that it does not rely on model. The pre-knowledge on the property of the dataset or making assumptions about the dataset is not required. Therefore, this approach is equally applicable to many datasets with different characteristics. However, there is one requirement for application of SFOER. As FDR for peptide identification is required during the optimization, SFOER can only process database search results performed with decoy database.

SFOER can also be easily extended to some special applications by slightly revision. Currently, SFOER only takes several SEQUEST scores such as Xcorr, ΔCn, Sp and Rsp as its weights. It was reported that some peptide properties obtained from the experiments of proteome analysis could be used to increase the confidence of peptide identifications. These properties including the pI values obtained from the isoelectric focusing (IEF)[41], hydrophobicity or elution times obtained from reversed phase LC separation (NET)[24], high accurate masses obtained from using of FT mass spectrometer[42] and so on. In principle, these properties as well as SEQUEST scores can be optimized simultaneously for filtering criteria by this software suite. And significant improvement in proteome coverage for proteome analysis is expected. Though SFOER was developed to optimize filtering criteria for SEQUEST database search, after slightly revision it should also be easily applied to the optimization of filtering criteria for other database search engines such as Mascot as long as the decoy database search strategy is applied.

## Conclusion

A software suite, named as SFOER, was developed using predictive genetic algorithm (GA) to optimize filtering criterion for SEQUEST database searching. The optimization was based on reversed database search where FDR can be easily determined. It was demonstrated that SFOER was able to maximize the number of identified peptides without increase of FDR. Compared with statistical approach – PeptideProphet, SFOER has nearly the same classification performance but cost much less processing time. Moreover, as it did not rely on possibly unfounded assumptions about the data, SFOER can create tailored criteria for datasets which are obtained from different samples, generated from different mass spectrometers, even searched with different database searching algorithms (weights need to be altered).

## Methods

### Materials and reagents

Magic C18AQ (5 μm, 100 Å pore size) was purchased from Michrom BioResources (Auburn, CA, USA), and Polysulfoethyl Aspartamide (5 μm, 200Å pore) was from PolyLC Inc (Columbia, MD, USA). PEEK tubing, sleeves, microtee and microcross were obtained from Upchurch Scientific (Oak Harbor, WA, USA). Fused-silica capillaries (50, 75 and 100 μm I.D.) were purchased from Polymicro Technologies (Phoenix, AZ, USA). All the water used in the experiment was purified using a Mill-Q system (Millipore, Bedford, MA, USA). Dithiothreitol (DTT), iodoacetamide were all purchased from Sino-American Biotechnology Corporation (Beijing, China). Urea, ammonium acetate, ammonium bicarbonate and acetic acid were obtained from Sigma (St. Louis, MO, USA). Trypsin was from Promega (Madison, WI, USA). Tris was from Amersco (Solon, Ohio, USA). Formic acid was obtained from Fluka (Buches, Germany). Acetonitrile (ACN, HPLC grade) was from Merck (Darmstadt, Germany). Protease inhibitor cocktail tablets (Complete Mini) were purchased from Roche.

### Sample preparation

Human blood plasma was obtained from one healthy male donor (age 37, O type), provided by Zhuanghe Blood Center (Dalian, China). An initial protein concentration of ~95 mg/mL was determined in plasma using Bardford method. Human liver tissue was homogenized in lysis buffer (40 mM Tris, 6 M guanidine HCl, 65 mM DTT, 310 mM NaF, 3.45 mM NaVO_3_, protease inhibitor cocktail) and then sonicated for 180 s followed by centrifugation at 25,000 g for 1 h. The supernatant was collected as protein sample and the concentration was determined by Braford assay.

The human plasma sample and human liver tissue lysate were reduced by DTT and alkylated by iodoacetamide. Then the solutions were diluted to 1 M guanidine-HCl, and pH values were adjusted to 8.1. Finally, trypsin was added (trypsin:protein, 1:50) and the protein samples were incubated at 37°C for 20 h. Tryptic digests were desalted with a C18 solid – phase cartridge.

Tryptic digests of standard proteins were prepared by digesting of 500 pmol reduced, iodoacetamide alkylated bovine serum albumin, horse myoglobin, horse cytochrome *c*, chick ovalbumin, human hemoglobin, bovine β-casein and bovine α-casein. Bovine serum albumin was purchased from Roche and all other standard proteins were from Sigma-Aldrich. These digests were pooled to prepare seven protein digest mixture. The final concentrations of these proteins were ranged from 16 to 300 fmol per microliter.

### LC-MS/MS analysis and database search

The configurations for 1D and 2D LC-MS/MS analysis were set as reported previously[34]. Therein, a Finnigan LTQ linear ion trap mass spectrometer (Thermo, San Jose, CA) was coupled with capillary reversed phase LC for collection of MS/MS spectra. The tryptic digest of 7 standard proteins was analyzed by 1D LC-MS/MS with 7 replicate runs and the Human sample digests were analyzed by 2D LC-MS/MS.

The acquired MS/MS spectra were searched using Turbo SEQUEST in BioWorks 3.2 software suite (Thermo Finnigan, San Jose, CA). For 7 standard proteins, database was the composite of protein sequences from yeast (9,492 entries) in forward and reverse orient as well as the forward and reversed sequences of all control proteins with trypsin and α-s2-casein (for the impurity of α-casein). The database used for two human proteome samples was a composite of normal IPI human database (v3.04, 49,078 entries) from European Bioinformatics Institute with reversed version of the same database attached in the end. MS/MS spectra were searched using fully tryptic cleavage constraints and up to two missed cleavage sites were allowed. Cysteine residues were set as static modification of +57.0215 Da and methionine residues were set as variable modification of +15.9949 Da. Mass tolerances were 2 Da for peptide and 1 Da for fragment. FDR was determined by Equation (1).

### Development of software suite SFOER using GA

A Java software suite named SFOER was developed to optimize filtering criteria using GA[29]. In GA, genes (SEQUEST scores for the criteria in this study) are generally encoded into binary character strings including only 0 and 1. Chromosome is composed of a single binary string where encoded genes are assembled one by one. Each chromosome in a generation is called an individual. For our GA, four cutoff values including Xcorr, ΔCn, Sp and Rsp were encoded into binary strings respectively. And chromosome which indicated filtering criterion was encoded into a 30-bit-long string. Details are shown in Table 4.

**Table 4:** Parameter settings for the genetic algorithm

		GA configuration
Variables		4
Population size		100
Crossover probability		0.2
Mutation probability		0.01
Bits	Xcorr	9
	ΔCn	9
	Sp	12
	Rsp	8
Fitness evaluation		n (peptides)

Definition of a fitness function for evaluating individual members of a population is perhaps the most crucial step in designing genetic algorithm. The goal in this study was to derive optimized filtering criteria that achieved maximal separation between correct and incorrect peptide identifications and generated maximum sensitivity for true positive peptide identifications under specified confidence level (e.g. >99%). However, in most proteome researches, numbers of total positive peptides were commonly unknown. Thus, we utilized the following fitness function:

F(p) = n(p),

where F(p) was the fitness value for a given filtering criterion which was consisted of several cutoff values for different scores, n(p) would be the number of overall positive peptide identifications passed this filtering criterion. And when FDR of peptide identifications filtered by a criterion was higher than specification, fitness of this criterion was set to zero. This function indicates the sensitivity of a specific criterion.

**Figure 6:**
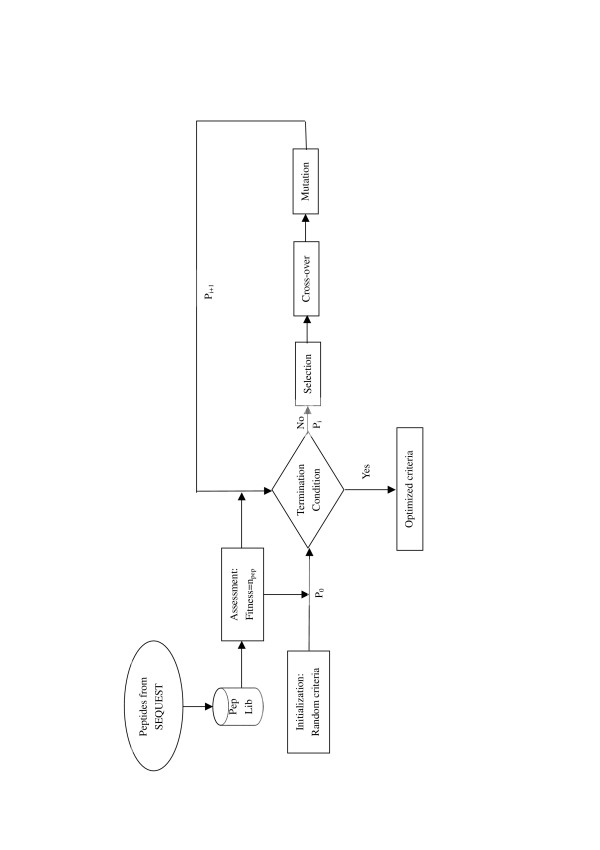
**Flowchart of the optimization procedure using genetic algorithm**. It starts with the initialization phase, which randomly generates the initial population P_0_. Population in the next generation P_i+1 _is obtained by applying genetic operators on current population P_i_. Fitness for each individual (criterion) is evaluated as the number of filtered peptides. Evolution continues until a terminating condition is reached. The selection, mutation and cross-over operator are used in genetic algorithm.

The genetic algorithm makes an optimization within a cycle of several stages. It includes creation of a population of individuals (criteria), evaluation of these individuals, selection of individuals and breeding aided by genetic manipulation to create offspring population (schematic shown in Figure 6):

1. Creation of the starting population: The starting point in genetic algorithm of the initial population was randomly generated. One complete chromosome was assembled of a certain number of different SEQUEST scores and the population size was set as 100.

2. Selection: Roulette wheel selection pattern was chosen for the determination of each individual's probability for reproduction and breeding, concerning the policy that the better a chromosome of a parent was the more descendants with the same chromosomes were reproduced. When the fitness of an individual became zero, this individual was selected as death, and replaced by a new initial individual.

3. Genetic manipulation: Two new breed chromosomes were then performed by a single-point cross-over, whereas genes were randomly altered along the length of a chromosome at one point according to a natural occurring cross-over. The cross-over rate was set to 0.2 and the rate of a subsequently performed point mutation, thus a binary character was changed from 1 to 0 or vice versa, was set to 0.01.

Steps 2, 3 were repeated until termination of the optimization. A stop criterion was not pre-defined, owing to limited data known about the search space. In this study, we used specific generations which can be set manually to terminate optimizations.

All database search results were processed by SFOER to generate optimized criteria on different confidence levels, and then peptide identifications were filtered by these sets of criteria. PeptideProphet which was downloaded as part of Trans-Proteomics Pipeline (TPP)[43] from The Seattle Proteome Center was also used to process these datasets. All peptides assigned from database searching were parsed by PeptideProphet to generate PeptideProphet-probability using default parameters. Manual adjustment of peptide probability threshold was used to generate peptide identifications with different confidence levels.

## Availability and requirements

The SFOER is developed using Java 2 Platform Standard Edition (J2SE) Development Kit 5.0 (Sun Microsystems, Inc) and is platform independent. Java Runtime Environment 1.5.0 or higher is required. It is distributed under a GNU General Public License (GPL) and is available at .

## Authors' contributions

X.N. Jiang carried out the study and developed the software implementing GA. H.F. Zou and M.L. Ye designed the whole project and helped to interpret data analysis results. X.G. Jiang and G.H. Han contributed to the sample preparation and analysis. All authors read and approved the final manuscript.

## Supplementary Material

Additional file 1 Distribution of peptides identified from human liver tissue lysate by SEQUEST. The data represented the detail information for the Xcorr ΔCn distribution of peptides identified from human liver tissue lysate by SEQUEST. Click here for file
