# Supplementary material for: Optimization of filtering criterion for SEQUEST database searching to improve proteome coverage in shotgun proteomics
Source: BMC Bioinformatics. 2007 Aug 31;8:323. doi: 10.1186/1471-2105-8-323 (PMC2040164; doi:10.1186/1471-2105-8-323)

Additional file 1: Distribution of peptides identified from human liver tissue lysate by SEQUEST

A) singly charged peptides

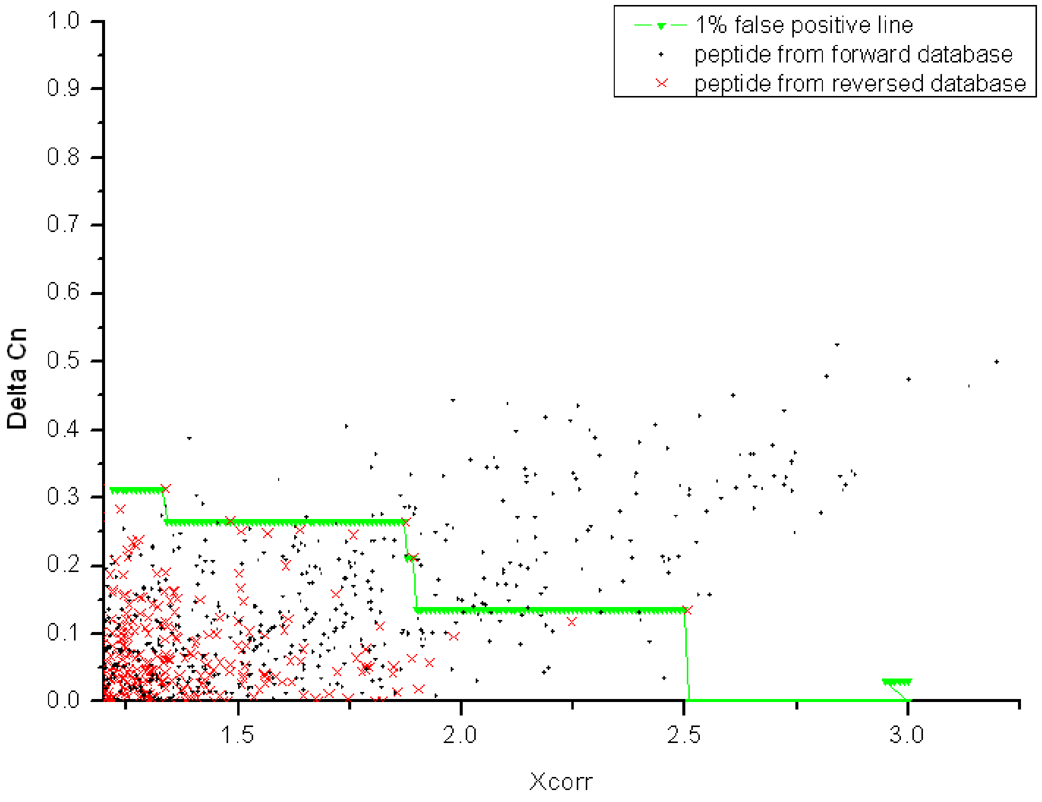

B) Doubly charged peptides

-

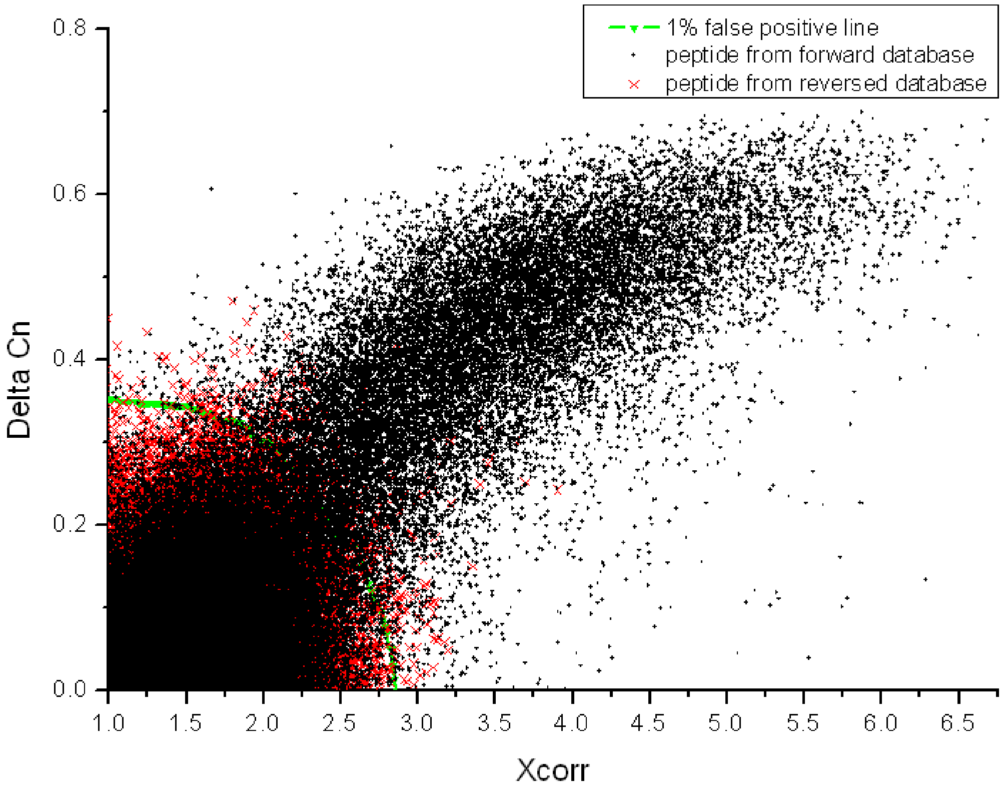

C) Triply charged peptides

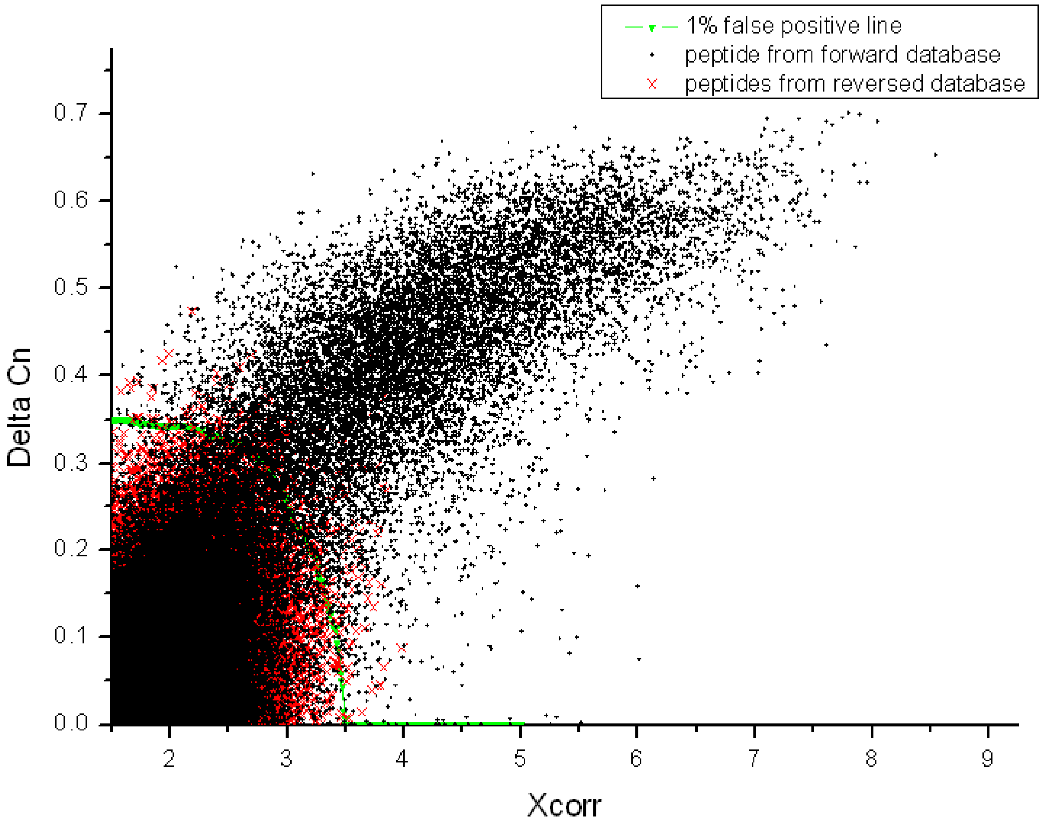

Supplement: Additional file 1 — Distribution of peptides identified from human liver tissue lysate by SEQUEST. The data represented the detail information for the Xcorr ΔCn distribution of peptides identified from human liver tissue lysate by SEQUEST. [file 1471-2105-8-323-S1.pdf]
